# Whole Transcriptome Sequencing Reveals How Acupuncture and Moxibustion Increase Pregnancy Rate in Patients Undergoing In Vitro Fertilization-Embryo Transplantation

**DOI:** 10.1155/2019/4179617

**Published:** 2019-05-28

**Authors:** Jie Cheng, Xun Jin, Jie Shen, Yanyun Mu, Qian Li, Liangjun Xia, Youling Gao, Youbing Xia

**Affiliations:** ^1^The Second Clinical College, Nanjing University of Chinese Medicine, Nanjing 210023, Jiangsu, China; ^2^School of Medicine and Life Sciences, Nanjing University of Chinese Medicine, Nanjing 210023, Jiangsu, China; ^3^The First Clinical College, Nanjing Medical University, Nanjing 211166, Jiangsu, China

## Abstract

**Background:**

In vitro fertilization and embryo transfer (IVF-ET) technology has been widely used in the therapy of refractory infertility. Previous studies showed that acupuncture can effectively increase the clinical pregnancy rate of IVF-ET. However, the molecular mechanism is unknown.

**Materials and Methods:**

In this study, we performed whole transcriptome sequencing for endometrial samples from infertile women who underwent acupuncture and moxibustion therapy or not. Differentially expressed noncoding RNAs (ncRNAs) and mRNAs were identified and their functions were predicted. Besides, a competitive endogenous RNA network was constructed to further interpret the molecular mechanism of acupuncture and moxibustion therapy on infecund patients. In addition, real-time PCR was applied to validate the RNA-seq results.

**Results:**

We identified 317 differentially expressed mRNAs and 82 ncRNAs in acupuncture and moxibustion therapy group compared with control group. Functional enrichment analysis suggested that these genes were significantly enriched in GO-BP terms associated with cellular transport, such as ATP hydrolysis coupled proton transport, vacuolar acidification, transferrin transport, and proton transport and metabolic process, including small molecule metabolic process and metabolic process. Pathway enrichment analysis enriched 11 terms, including oxidative phosphorylation, synaptic vesicle cycle, mineral absorption, and metabolic pathways. Four of five selected differentially expressed genes were validated by real-time PCR.

**Conclusion:**

Our results suggested that acupuncture and moxibustion therapy might increase the pregnancy rate of patients undergoing IVF-ET by the regulation of ncRNAs.

## 1. Introduction

Currently, the number of infertile patients continues to increase, with estimated global infertility rates of 8-12% and an average of 9 % for couples of childbearing age [1]. During the past decades, the assisted reproductive technology (ART), such as In vitro fertilization and embryo transfer (IVF-ET) and artificial insemination, underwent great progression and therefore, the pregnancy rates have been improved significantly worldwide [2]. Nevertheless, the probability of implantation failure after ET is nearly 60% to 70%.

Nowadays, traditional Chinese medicine (TCM), including Chinese herbal medicine and acupuncture along with moxibustion therapy and massage, is being increasingly utilized for treating gynecological health disorders [3]. Acupuncture is the Chinese traditional way of treatment which involves any insertion of needles at certain pressure points of the body. Studies have shown that acupuncture applied on the lower limbs and in the lower abdomen can increase blood flow to the ovaries and uterus and can thus increase the thickness of endometrial lining [4, 5]. However, seldom researches have been conducted to investigate the effect of acupuncture and moxibustion therapy on pregnancy outcome of IVF-ET at molecular level.

With the development of sequencing technologies, a multitude of noncoding RNA (ncRNA) species has been widely discovered, including microRNAs (miRNAs), circular RNAs (circRNAs), and long ncRNAs (lncRNAs) [6]. LncRNA is usually expressed at a lower level, while showing more cell type-specific expression patterns compared to protein-coding genes [7]. Geisler and Coller illuminated the action of lncRNA which can be catalogued into two groups: lncRNAs function as regulator of transcription by enhancer RNAs (eRNAs) or cis- or transregulation; lncRNAs function as regulator of transcription [8]. Moreover, lncRNA has been shown to play important role in various biological processes and diseases, including immune [9], development [10], and cancer [11]. Recent study showed that lncRNA might be involved in infertile women with endometriosis [12]. However, the roles of lncRNA in pregnancy rate for IVF-ET are still unknown and whether lncRNA acts in the acupuncture and moxibustion therapy remains to be demonstrated.

In this study, we performed whole transcriptome sequencing for endometrial samples from infertile women who underwent acupuncture and moxibustion therapy or not. Differentially expressed noncoding RNAs and mRNAs were identified and their functions were predicted. Besides, a competitive endogenous RNA network was constructed to further interpret the basic underlying mechanisms by which acupuncture and moxibustion therapy increase the pregnancy outcome for IVF-ET. We anticipate our results which could be helpful for understanding the role of acupuncture in treating women's health.

## 2. Materials and Methods

### 2.1. Patients

The endometrial samples were collected from infertile women at childbearing age under embryo transplantation at our hospital from January 2016 to June 2017. All women experienced infertility for 1.5 to 9 years because of salpingitis, polycystic ovarian syndrome (PCOS), or diminished ovarian reserve (DOR). A total of 12 women who planned to receive IVF-ET were recruited into this study. The women were assigned into case group (n = 6) or control group (n = 6) according to patients' characteristics or response during previous cycles. IVF for women in both groups were performed as previously described [13]. For women in case group, acupuncture and moxibustion at points of* guanyuan*,* zigong*,* zusanli*,* sanyinjiao*,* shenshu, *and* ciliao *were also performed. The endometrium thickness (EMT) was measured at the maximal distance between each myometrial-endometrial interface by using vaginal ultrasonography in the mid-luteal phase. This study was approved by the ethics committee of Nanjing medical University (No. (2014)204). All women provided written informed consent.

### 2.2. Acupuncture and Moxibustion Therapy Procedure

For the case group, women received acupuncture and moxibustion from the first day of two menstrual cycles before entering IVF-ET to the day of IVF-ET once every other day. The procedure was as follows: women rested in a supine position. Needles with length ranging from 25 mm to 40 mm were inserted and mild reinforcing and attenuating techniques were applied after arrival of qi with manipulating of needle for 30s, twirling of needle for 90°, lifting-thrusting rage of 2 mm, and frequency of 60-100 times/min. Needle retention time was 30 minutes at each acupuncture point. During needle retention, the Han's acupoint neurostimulator (HANS, LH202) was connected to the needles with* ciliao-ciliao* and* zigong-zigong*. The frequency of HANS was 2/15 Hz and strength was depending on patients' comfort. Moxa-moxibustion was applied to* guanyuan* and* zusanli* as follows: the moxa stick was hanged vertically at 2 cm above particular point for 10 min at each point. The moxa stick was raised if the patients cannot tolerate.

### 2.3. RNA Extraction, cDNA Library Construction, and Sequence Analysis

Endometrial samples were obtained during the mid-luteal phase of the menstrual cycle. Total RNAs from the endometrial samples of the two groups were extracted by using TRIzol reagent (Invitrogen, Carlsbad, CA, USA). Nanodrop 2000 spectrophotometer (Wilmington, DE, USA) was used to measure the quantity and quality of the extracted RNA. The qualified RNA samples with A260/280 > 1.9 were used for cDNA library construction as described previously [14]. Whole transcriptome sequencing was performed on HiSeq™ 2500 (Illuminainc, San Diego, CA, USA) in 150 bp paired-end reads. The raw reads were qualified using Fast-QC(v0.11.7) (http://www.bioinformatics.babraham.ac.uk/projects/fastqc/) by filtering the empty reads, the adaptor sequences, and the sequences with low quality (> 50% of bases whose Q scores were ≤ 10%). The clean data were mapped to human reference genome (GRCh38) using HISAT2 [15]. The abundance of gene expression was calculated using fragments per kilobase of exon per million fragments mapped (FPKM). The differentially expressed genes (DEGs, including mRNAs and ncRNAs) in the two groups were screened by DESeq [16] based on the criteria of |log_2_⁡  fold  change  (FC)| > 1 and false discovery rate (FDR) < 0.05.

### 2.4. Functional Enrichment Analysis

The function of the DEGs was predicted by Gene Ontology (GO) analyses (http://www.geneontology.org) in three categories: cellular component (CC), molecular function (MF), and biological process (BP)[17]. Kyoto Encyclopedia of Genes and Genomes (KEGG) was performed to identify the cellular signal pathways that the DEGs involved in. The threshold of significance for GO and KEGG analyses was still defined by FDR < 0.05.

### 2.5. miRNA Prediction and Construction of CeRNA Network

The miRNA candidates that target the DEGs were predicted using miRanda and RNAhybrid [18, 19]. The predicted diameters were set to energy < -30 and score >160 for miRanda prediction and energy < -30 for RNAhybrid prediction. Only the overlapped miRNAs predicted by the two databases were retained for further analysis. Cytoscape V3.6. 0 was used to construct the CeRNA network.

### 2.6. Validation of the DEGs

The expression levels of three key differentially expressed mRNAs (DEmRNAs) and three key differentially expressed lncRNAs (DElncRNAs) were validated by real-time PCR. Total RNA from endometrial samples was extracted by using TRIzol reagent (Invitrogen, Carlsbad, CA, USA). Complementary DNA (cDNA) was synthesized using the PrimeScript RT kit (Takara, Dalian, China). qRT-PCR was performed with a SYBR-Green PCR kit (Roche Diagnostics, Indianapolis, IN, USA) via a StepOnePlus Real-Time PCR system (Applied Biosystems, Foster City, CA, USA). The sequences of the primers used for the PCR are presented in Table 1. The PCR cycling conditions were as follows: 95°C for 10 min, 45 cycles of 95°C for 15 sec, 60°C for 60 sec, dissociation at 95°C for 10 sec, 60°C for 1 min, and 95°C for 15 sec. The results were analyzed using the 2^−ΔΔCt^ method with internal control of B2M gene. The qRT-PCR reactions were all repeated three times.

### 2.7. Statistical Analysis

RNA sequence data were analyzed by bioinformatics methods as indicated above. Other data were analyzed by SPSS 21.0 (IBM, Chicago, USA). Differences between two groups were compared by two-tailed Students'* t*-test and P < 0.05 was considered as statistically significant.

## 3. Results

### 3.1. Acupuncture and Moxibustion Treatment Increased the Pregnancy Rate of IVF-ET

The characteristic of women in the two groups is shown in Table 2. The age and infertility years of women in case group was significantly higher than that in control group (*P *= 0.03 and* P *= 0.02, respectively). The other characteristics including BMI, age of menarche, menstrual cycle, menstrual duration, and EMT before acupuncture and moxibustion were comparable between these two groups (P > 0.05). The EMT was decreased significantly in the patients with acupuncture and moxibustion compared to those without acupuncture and moxibustion treatment (P < 0.05), while the EMT in patients has no significant difference between before and after treatment of acupuncture and moxibustion (P > 0.05). Importantly, the pregnancy rate in the women treated with acupuncture and moxibustion (83.33%) was higher than that in the women who did not receive this treatment (50.00%).

### 3.2. Identification of DEGs

**Figure 1:**
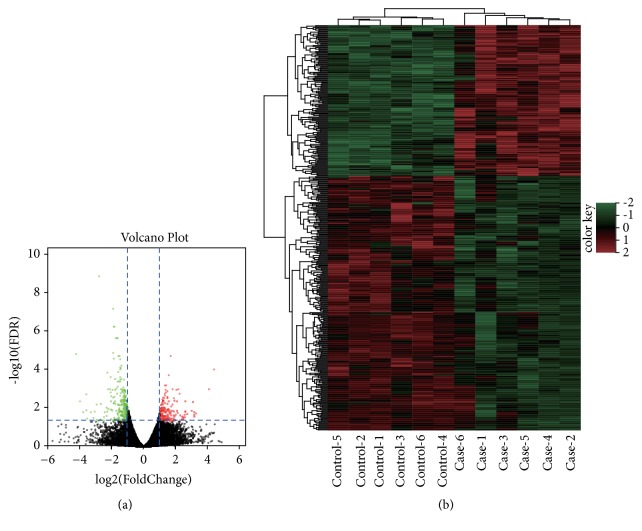
Differentially expressed (DE) lncRNAs and mRNAs between women received acupuncture and moxibustion (case group) or not (control group). (a) The volcano figure analysis of differentially expressed (DE) lncRNAs and mRNAs in case and control groups of patients by RNA-seq. The red, green, and grey colors stand for the terms means upregulation downregulation and normal expression, respectively. (b) Hierarchical cluster analysis of differentially expressed (DE) lncRNAs and mRNAs in control groups and case groups by RNA-seq. The red and green colors stand for the terms mean upregulation and downregulation, respectively.

Based on the criteria of |log_2_  ⁡FC| >1 and FDR < 0.05, a total of 317 differentially expressed mRNAs (including 95 upregulated mRNAs and 222 downregulated mRNAs) and 82 ncRNAs (including 50 upregulated ncRNAs and 32 downregulated ncRNAs) were screened out in case group compared with control group (Figure 1(a)). Hierarchical cluster analysis suggested these DEGs (including DEmRNAs and DEncRNAs) could significantly separate the samples into control groups and case groups (Figure 1(b)), suggesting the reliability of our analysis. The top 10 DEmRNAs and DEncRNAs were shown in Table 3.

### 3.3. Functional Analysis of the DEGs

**Figure 2:**
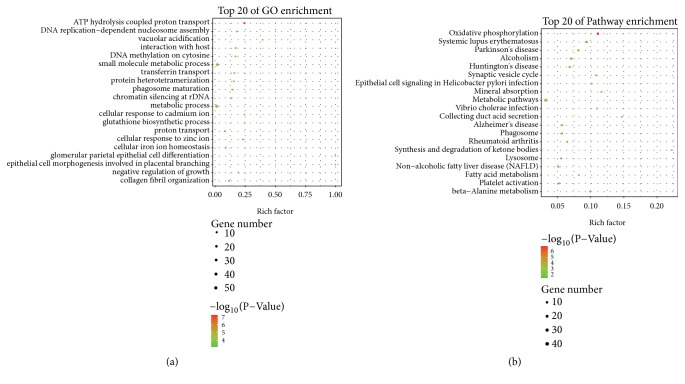
Gene Ontology (GO) and Kyoto Encyclopedia of Genes and Genomes (KEGG) pathway analysis results. The top 10 enrichment of GO (a) and KEGG (b) terms for DEmRNAs in the ceRNA network. C: DEmRNAs enriched in the top 10 pathway terms. Red stands for significant terms (P-value < 0.05). Green stands for nonsignificant terms (P-value > 0.05).

In order to further interpret the DEGs, we performed GO and pathway enrichment analysis for the DEGs. A total of 19 GO-BP terms were significantly enriched based on the criteria of FDR < 0.05. Most of these enriched GO-BP terms were related to cellular transport, such as ATP hydrolysis coupled proton transport (FDR = 8.001e-05), vacuolar acidification (FDR = 0.006), transferrin transport (FDR = 0.007), and proton transport (FDR = 0.018) and metabolic process, including small molecule metabolic process (FDR = 0.006) and metabolic process (FDR = 0.011) (Figure 2(a)). Pathway enrichment analysis enriched 11 terms based on the cut-off value of FDR < 0.05, including oxidative phosphorylation (FDR = 2.769e-05), synaptic vesicle cycle (FDR = 0.014), mineral absorption (FDR =0.016), and metabolic pathways (FDR = 0.016) (Figure 2(b)).

### 3.4. CeRNA Network

**Figure 3:**
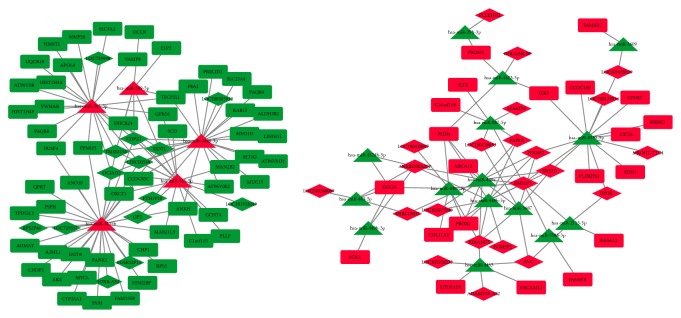
lncRNA-miRNA-mRNA network. The red color stands for upregulation and the green color represents downregulation.

By merging the relationships among DEncRNAs, DEmRNAs, and the overlapped predicted miRNAs, two ceRNA networks were constructed using Cytoscape (Figure 3): one network constructed with upregulated miRNAs and downregulated mRNAs and lncRNAs; the other one consisting of downregulated miRNAs and upregulated mRNAs and lncRNAs. In these networks, hundreds of ceRNA relationships were predicted. For example, lncRNA TMEM154 might function as a ceRNA to regulate MMP26/ATP6V0B/HIST1H4A by sponging hsa-miR-345-3p. Besides, this lncRNA might also regulate the expression of CYP26A1/FAM155B/MYCL by sponging hsa-miR-3135b. In the downregulated miRNA network, lncRNA ADAMTS9-AS2 might regulate expression of DSCAML1 and LDLRAD1 by sponging hsa-miR-4463. The lncRNA MROH7-TTC4 might regulate the expression of IER3 and EDN1 by sponging hsa-miR-6510-5p.

### 3.5. Validation of DEGs

**Figure 4:**
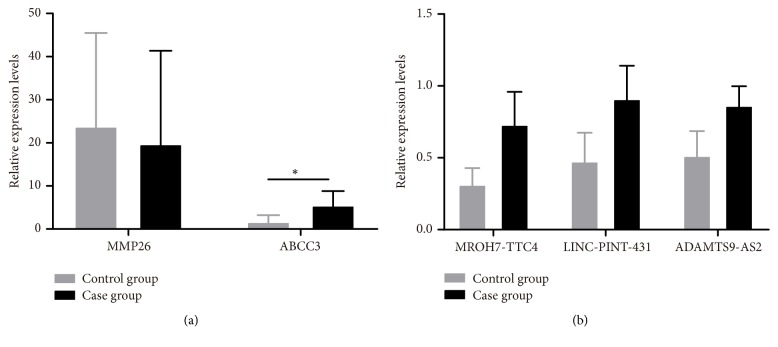
The validation of expression on selected DEmRNAs (a) and associated lncRNA (b) in endometrial tissue of case group and control group by RT-PCR. *∗* refers to the statistically significant difference (P < 0.05).

We further selected 2 DEmRNAs and 3 DElncRNAs to perform real-time PCR to further validate our results. As shown in Figure 4, downregulation of MMP26 and upregulation of ABCC3 in RNA-seq results were validated by real-time PCR, however, the difference of MMP26 between case group and control group did not reach statistical significance (P > 0.05). Besides, the 3 upregulated lncRNAs in case group were all validated by real-time PCR (*P*< 0.05).

## 4. Discussion

Previous study showed acupuncture and moxibustion affect the estrogen level in HCG days and improve the endometrial receptivity, thus improving the high quality embryo rate, indicating acupuncture and moxibustion might be an adjuvant therapy to improve the outcome of IVF-ET [20]. However, the molecular mechanism involved in this effect has not been elucidated. In this study, we identified 317 differentially expressed mRNAs (including 95 upregulated mRNAs and 222 downregulated mRNAs) and 82 ncRNAs (including 50 upregulated ncRNAs and 32 downregulated ncRNAs) in acupuncture and moxibustion therapy group compared with control group. Functional enrichment analysis suggested that these genes were significantly enriched in GO-BP terms associated with cellular transport, such as ATP hydrolysis coupled proton transport, vacuolar acidification, transferrin transport, and proton transport and metabolic process, including small molecule metabolic process and metabolic process. Corresponding to GO results, pathway enrichment analysis enriched 11 terms, including oxidative phosphorylation, synaptic vesicle cycle, mineral absorption, and metabolic pathways.

Based on the DEGs list and the CeRNA network, we selected 5 DEGs for validation, including 2 DEmRNAs (MMP26 and CYP26A1) and 3 DElncRNAs (MROH7-TTC4, LINC-PINT-431, and ADAMTS9-AS2). MMP26 (matrix metalloproteinase 26) is a member of the matrix metalloproteinase family. It is involved in the breakdown of extracellular matrix in normal physiological processes, such as reproduction, embryonic development, and tissue remodeling. The role of MMP26 in IVF-ET is controversial. It is believed that MMPs are essential for embryo attachment and the following invasion in endometrium. MMP26 plays important roles in degrading extracellular matrix, such as fibronectin, type IV collagen, and insulin-like growth factor-binding protein 1, and involves endometrium remodeling [21]. Qiao* et al.* found that expression of MMP26 was significantly inhibited in patients with PCOS during window of implantation. However, there are also studies which demonstrated that MMP26 was significantly increased in women with unexplained infertility compared with fertile women [22]. Besides, upregulation of MMPs has also been associated with implantation abnormalities and inflammatory milieu in endometrium [23], and suppression of MMPs is necessary for endometrial stability [24]. Our study showed that expression of MMP26 is decreased 4.27-fold in case group compared with control group in RNA-seq results. In the validation by real-time PCR, the expression of MMP26 was also decreased in case group compared with control group, though it not reached to statistical significance. Our study indicated that acupuncture and moxibustion might increase endometrial stability and aid in pregnancy outcome by downregulating MMP26.

ABCC3 (ATP-binding cassette subfamily C member 3) is a member the superfamily of ATP-binding cassette (ABC) transporters. ABC proteins transport various molecules across extra- and intracellular membranes. In this study, ABCC 3 was enriched in GO-BP terms of ATP catabolic process and was significantly upregulated after acupuncture and moxibustion therapy. Previous studies have shown that acupuncture applied on the lower limbs and in the lower abdomen can increase blood flow to the ovaries and uterus and can thus increase the thickness of endometrial lining [4, 5]. The process of blood flow is involved in ATP consumption. Therefore, we speculated that acupuncture and moxibustion therapy applied on the points of* guanyuan*,* zigong*,* zusanli*,* sanyinjiao*,* shenshu, *and* ciliao *can increase blood flow by increase ATP catabolic process and thus increase endometrial receptivity and improve the pregnancy outcome of IVF-ET.

In conclusion, our results suggested that acupuncture and moxibustion therapy applied on the points of* guanyuan*,* zigong*,* zusanli*,* sanyinjiao*,* shenshu, *and* ciliao *might be useful in increasing the pregnancy outcome for IVF-ET by regulation of lncRNA.

## Figures and Tables

**Table 1:** Primers sequences for qRT-PCR.

Gene	Direction	Sequence
MMP26	Forward	5′-TGGAGCAATGTGACCCCTT-3′
	Reverse	5′-GCCCACTGCCAGAAAGAAAC-3′
CYP26A1	Forward	5′-CAGGCACTAAAGCAATCTTCAAC-3′
	Reverse	5′-GGTAGAGCCCCAGGTAAGTGAT-3′
NQO1	Forward	5′-GAAAGGATGGGAGGTGGTGG-3′
	Reverse	5′-CAGACTCGGCAGGATACTGAAAG-3′
MROH7-TTC4	Forward	5′-CTGTGGACTGTGCAGTAAGTGTTG-3′
	Reverse	5′-GCAGAAAGAAATCAAGTCAGGGA-3′
LINC-PINT-431	Forward	5′-TGTAACAGCTGAGAGGAAAATGGA-3′
	Reverse	5′-CCTCATTTTTCCTGTTCAGTGGT-3′
ADAMTS9-AS2	Forward	5′-CCATCAAAGGGAAAAACACACAT-3′
	Reverse	5′-ATGAGTTTGGGCACGCAGTT-3′

**Table 2:** General characteristic of women in this study.

Terms	Case group (n=6)	Control group (n=6)	*P*
Age, years	31±2.37	27.5 ±2.43	0.03
Infertility years	5.75±2.40	2.83±0.68	0.02
BMI, kg/m^2^	21.52±1.35	20.77±1.01	0.30
Age of menarche, years	14.33±1.51	13±0.89	0.09
Menstrual cycle, days	58.75±60.04	32.08 ±10.10	0.30
Menstrual duration	6.58±0.86	5.58±1.16	0.12
EMT before acupuncture and moxibustion (on the day of transplant)	9.3±1.15	10.26±1.34	0.21
EMT after acupuncture and moxibustion (on the day of transplant)	8.65±0.73	10.26±1.34	0.02
Clinical pregnancy rate	83.33%	50%	-

*∗* BMI= body mass index; EMT= endometrium thickness. Difference between the case and control groups was compared by student's test. *P*< 0.05 indicated statistical significance.

**Table 3:** The top 10 differentially expressed mRNAs and top 10 differentially expressed ncRNAs.

ID	Log2FC	FDR	Style	Type
MUC5AC	4.05	0.001	UP	mRNA
HTR6	3.28	0.019	UP	mRNA
FAM135B	3.15	0.022	UP	mRNA
C9orf50	3.144	0.028	UP	mRNA
CDHR2	3.124	0.016	UP	mRNA
ADAM21	-6.08	0.001	DOWN	mRNA
MMP26	-4.27	1.56E-05	DOWN	mRNA
KRT31	-4.04	0.004	DOWN	mRNA
SFRP2	-3.84	0.016	DOWN	mRNA
FXYD4	-3.60	0.002	DOWN	mRNA
SSXP10	4.37	9.30E-05	UP	ncRNA
LOC101929039	2.70	0.021	UP	ncRNA
LOC101927940	2.60	0.015	UP	ncRNA
LOC101928233	2.58	0.004	UP	ncRNA
LINC01091	2.46	0.036	UP	ncRNA
GP2	-3.64	0.013	DOWN	ncRNA
LOC101930644	-3.58	0.035	DOWN	ncRNA
LOC100505912	-3.17	0.034	DOWN	ncRNA
RNA5SP324	-2.86	0.047	DOWN	ncRNA
TMEM154	-1.99	0.001	DOWN	ncRNA

## Data Availability

The data used to support the findings of this study are available from the corresponding author upon request.
